# Supplementary material for: An intergenerational lipid memory of the social environment in C. elegans
Source: bioRxiv. 2025 Jun 3:2025.06.03.657568. Preprint. [Version 1] doi: 10.1101/2025.06.03.657568 (PMC12157553; doi:10.1101/2025.06.03.657568)
Supplement: 2 [file NIHPP2025.06.03.657568v1-supplement-2.pdf]

bioRxiv preprint doi: https://doi.org/10.1101/2025.06.03.657568; this version posted June 3, 2025. The copyright holder for this preprint (which was not certified by peer review) is the author/funder, who has granted bioRxiv a license to display the preprint in perpetuity. It is made available under aCC-BY 4.0 International license.

Figure S1

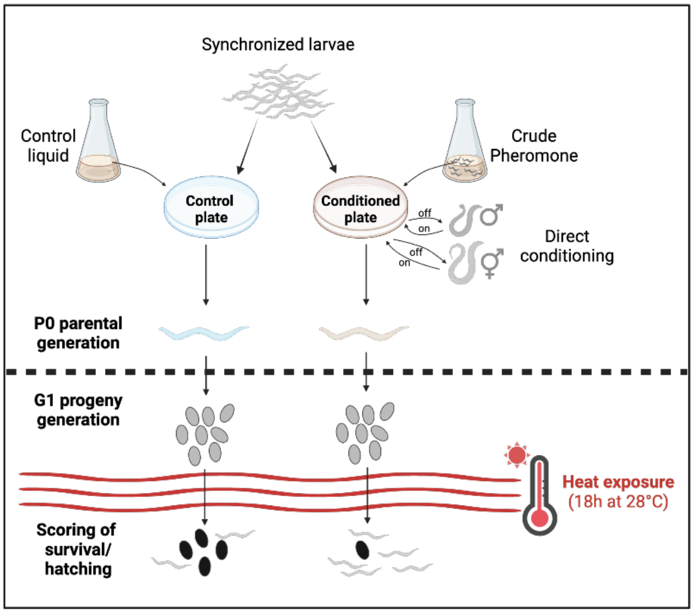

bioRxiv preprint doi: https://doi.org/10.1101/2025.06.03.657568; this version posted June 3, 2025. The copyright holder for this preprint (which was not certified by peer review) is the author/funder, who has granted bioRxiv a license to display the preprint in perpetuity. It is made available under aCC-BY 4.0 International license.

Figure S2

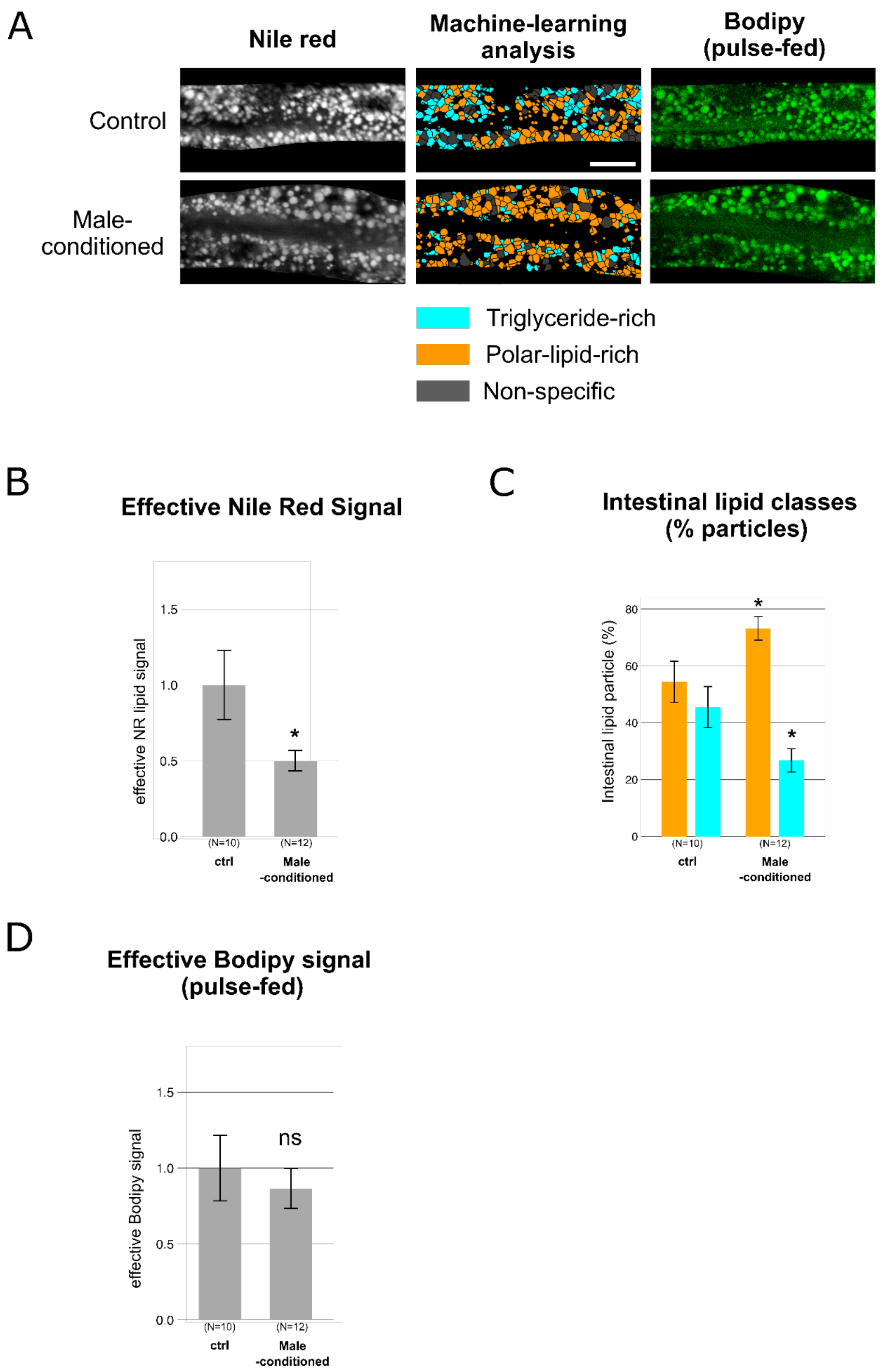

bioRxiv preprint doi: https://doi.org/10.1101/2025.06.03.657568; this version posted June 3, 2025. The copyright holder for this preprint (which was not certified by peer review) is the author/funder, who has granted bioRxiv a license to display the preprint in perpetuity. It is made available under aCC-BY 4.0 International license.

Figure S3

A

mothers fed with bacteria expressing *pos-1* dsRNA

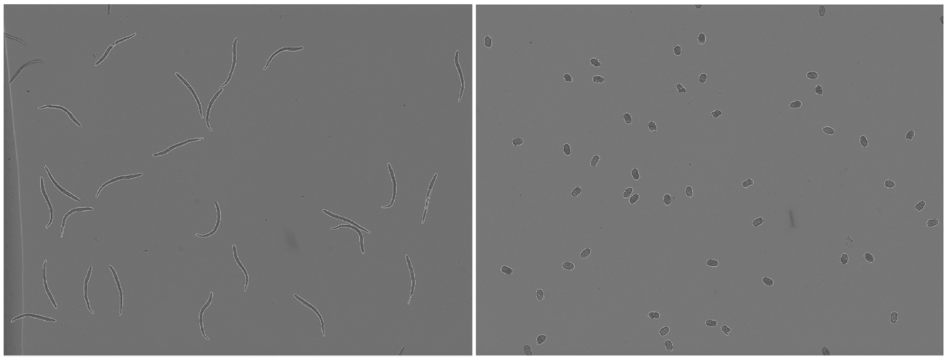

sid-1(q9) ; vha-6p::sid-1 **wildtype (N2)**
